# Validation of the 10-item Chinese perceived stress scale in elderly service workers: one-factor versus two-factor structure

**DOI:** 10.1186/2050-7283-1-9

**Published:** 2013-06-19

**Authors:** Siu-man Ng

**Affiliations:** Department of Social Work and Social Administration, The University of Hong Kong, Hong Kong SAR, China

**Keywords:** Stress, Measure, Scale validation, Psychometrics

## Abstract

**Background:**

Despite its popularity, the psychometric properties of the 10-item Chinese Perceived Stress Scale (CPSS-10) in working adults are yet to be evaluated.

**Methods:**

This study examined CPSS-10 in elderly service workers through a questionnaire survey. The sample was randomly split into two for exploratory (EFA) and confirmatory factor analysis (CFA).

**Results:**

A high response rate (93%) was achieved, resulting in 992 completed questionnaires. EFA with the first split sample favored a two-factor over a one-factor solution. The second factor had eigenvalue 2.00 and provided 19.95% explained variance. In CFA with the second split sample, the two-factor structure showed satisfactory goodness-of-fit (CFI = 0.93, RMSEA = 0.06) while the one-factor structure showed poor data fit (CFI = 0.62, RMSEA = 0.14). Further analyses on the two-factor structure revealed that the whole scale and two subscales had acceptable internal consistency (Cronbach’s alphas = 0.67 to 0.78). The total score was positively associated with perceived workload and burnout (*r* = 0.17 to 0.48), but negatively with work engagement (*r* = −0.13 to −0.30). In contrary to previous studies, a low inter-factor correlation (*r* = −0.08) was revealed.

**Conclusions:**

CPSS-10 showed a stable two-factor structure with satisfactory internal consistency and construct validity.

## Background

Because of its extensive associations with health outcomes, stress has long been an important research topic. The transactional model regards stress as an interaction between the individual and the environment (Lazarus & Folkman 1984). Stress arises when one appraises an event as threatening to the accomplishment of important goals or overwhelming to one’s resources. The transactional meaning of stress thus not only incorporates environmental and personal characteristics, but also emphasizes the subjective appraisal of the event. The same stimulus may generate different interpretations, responses, and coping strategies among individuals with different experiences and personality traits.

In accordance with the transactional model of stress, Cohen, Kamarck, and Mermelstein (Cohen et al. 1983) developed the Perceived Stress Scale as a global stress measure. The scale assesses the degree to which situations are appraised as stressful during the previous month. Originally, this self-report scale comprised 14 items. Later the authors reported the shortened 10-item version (PSS-10) as psychometrically superior to the original 14-item version (Cohen & Williamson 1988). The PSS-10 has demonstrated adequate internal consistency, with Cronbach’s alpha coefficients ranging from 0.72 to 0.91 in previous studies conducted in samples of college students, participants of smoking-cessation program, adults in the community, workers of occupational health care centers, policewomen, medical students and hospital inpatients (Leung et al. 2010; Orucu & Demir 2009; Wongpakaran & Wongpakaran 2010). Previous research has also found evidence for the scale’s construct validity. It was negatively correlated with positive measures, such as perceived health in college students and participants of smoking-cessation program, and self-esteem of medical students and hospital inpatients. On the other hand it was positively correlated with negative measures, such as health complaints in college students (Otto et al. 2004), state anxiety and depressive symptoms in depression patients, medical students and hospital inpatients (Orucu & Demir 2009; Wongpakaran & Wongpakaran 2010), susceptibility to the common cold in healthy adults (Cohen et al. 1993), and emotional exhaustion in college students (Ramirez & Hernandez 2007).

Regarding the scale’s dimensionality, most researchers have found evidence for a two-factor structure (Eskin & Parr 1996; Wang et al. 2011; Wongpakaran & Wongpakaran 2010; Orucu & Demir 2009; Otto et al. 2004; Roberti et al. 2006; Reis et al. 2010). The two factors revealed in the EFAs were named Perceived Helplessness (comprised of items 1, 2, 3, 6, 9, and 10) and Perceived Self-efficacy (comprised of items 4, 5, 7, and 8, which are reversely coded when computing the total score).

In a study of 60 suicide survivors, Mitchell, Crane, and Kim (Mitchell et al. 2008) argued for a one-factor structure for PSS-10 even though the eigenvalue of the second factor was still bigger than 1. The main arguments were, firstly, the primary factor already accounted for sufficient amount of the total variance explained (56.6%), and secondly, being conceptualized as a global assessment of stress, a one-dimensional structure for PSS-10 is theoretically more coherent.

PSS, especially the 10-item version, has been widely adopted in health outcome studies. The PSS-10 has been translated and validated in Japanese (Mimura & Griffiths 2008), Swedish (Eskin & Parr 1996), Spanish (Ramirez & Hernandez 2007), Turkish (Otto et al. 2004), Portuguese (Reis et al. 2010), French (Lessage et al. 2012), and Thai (Wongpakaran & Wongpakaran 2010). Given the close relation between stress and a wide range of well-being measures such as perceived health (Cohen et al. 1983), negative affect (Cohen et al. 1993; Ho et al. 2004), and workplace well-being (Duran et al. 2006; Prosser et al. 1997; Ro et al. 2010), it is essential to have a measure of perceived stress validated in the Chinese people. Although the PSS-10 has been translated into Chinese (Lee & Crockett 1994) and the Chinese PSS-10 (CPSS-10) has been applied in various previous studies (Ho et al. 2004; Gao et al. 2009; Chung & Tang 2006), the psychometric properties of CPSS-10 have rarely been examined. These previous studies have merely reported the scale’s internal consistency (Cronbach’s alphas ranging from 0.74 to 0.82), and provided minimal information on its factorial and construct validity. The only exceptions were more recent studies by Leung (Leung et al. 2010) and Wang (Wang et al. 2011). Leung examined the factor structure, internal consistency and construct validity of the 4, 10 and 14-item versions of CPSS. Leung concluded that the 10-item version CPSS, with a two-factor structure, showed the best overall psychometric properties. Wang evaluated the factor structure of CPSS-10 and recommended a 2-factor structure. However, since these two studies were on very specific groups, cardiac patients who smoked and policewomen respectively, the transferability of the findings to other population groups is unsure.

Because of the high relevance of the notion of stress in workplace, it is essential to validate CPSS-10 in working adults. The current study evaluated the psychometric properties of CPSS-10 in elderly service workers in Hong Kong.

## Methods

### Participants

Participants were part of a cross-sectional questionnaire study of occupational well-being carried out among the workers of elderly service units of a large social service organization in Hong Kong (Ng et al. 2011). All potential participants were invited to participate in the study by an individual invitation letter which included introduction of the study, consent form and a set of questionnaire. They were informed of the survey’s aim and assured that their responses would be kept strictly anonymous and confidential. Voluntary participation was ensured throughout the study and written informed consent was collected from each participant. Each completed questionnaire was put into an envelope and then sealed by the participant him/herself. All completed questionnaires in sealed envelopes were sent to the research team for data processing. The elderly service agency had no access to the completed questionnaires. Ethical approval for the study was granted by the Human Research Ethics Committee for Non-Clinical Facilities of the The University of Hong Kong (reference number EA171210). A high response rate (93.0%) was resulted, leading to a sample of 992 workers participated in the study. Table 1 shows the demographic characteristics of the study participants. The mean age of the sample was 43.2 years (*SD* = 10.2). About five-sixths of the participants (83.5%) were women, and 16.5% men. Most participants (68.5%) were married, 22.6% were single, and 9.0% were divorced. With respect to the highest level of education attained, 14.2% of the participants attained primary education, 38.5% attained junior secondary education, 28.6% attained senior secondary education, and 18.7% attained tertiary education. The mean years of service in the elderly service organization was 7.9 years (*SD* = 6.7).

**Table 1:** **Characteristics of participants in the study**

	Whole sample	Split sample 1	Split sample 2		
Variable	(n = 992)	(n = 491)	(n = 501)		
	%	%	%	***χ*** ^***2***^	***p***
Gender					
Male	16.5	15.5	17.5	*0.72*	*0.40*
Female	83.5	84.5	82.5		
Marital status					
Single	22.6	20.6	24.4	*2.33*	*0.31*
Married	68.5	69.7	67.3		
Divorced	9.0	9.7	8.3		
Educational level					
Primary	14.2	15.4	13.1	*6.85*	*0.08*
Junior secondary	38.5	41.5	35.7		
Senior secondary	28.6	25.3	31.8		
Tertiary	18.7	17.9	19.5		
Perceived workload					
Low	0.9	1.4	0.4	*6.88*	*0.14*
Normal	47.8	46.2	49.8		
High	40.8	43.2	38.5		
Very high	10.5	9.1	11.7		
	*M (SD)*	*M (SD)*	*M (SD)*	*t*	*p*
Age – years	43.2 (10.2)	43.6 (10.2)	42.9 (10.3)	*0.95*	*0.35*
Job tenure - years	7.9 (6.7)	7.8 (6.9)	7.9 (6.5)	*0.25*	*0.81*

### Measures

To assess its psychometric properties, we administered the CPSS-10 together with a battery of validation scales to the study participants (Lee & Crockett, 1994). Among the 10 items of the CPSS-10, six items are negatively worded (e.g., “How often did you feel that you were unable to control important things in your life?”) and the remaining four are positively worded (e.g., “How often did you feel that you were on top of things?”). The same response format is adopted in the CPSS-10 as in the original PSS, and the items are rated in a 5-point Likert response format (0 = *never* to 4 = *very often*). When computing the total score, the four positive items are reversely coded and then added to the six negative items, so that a higher total score denotes greater perceived stress.

To evaluate the construct validity of the CPSS-10 in working adults, we incorporated the Maslach Burnout Inventory – General Survey (MBI-GS), the Utrecht Work Engagement Scale (UWES), and a single item inquiring perceived workload into the questionnaire. The MBI–GS is a 16-item self-report scale widely used to measure burnout through the three dimensions of Emotional Exhaustion (exhaustion of passion, enthusiasm, and empathy), Cynicism (negative and apathetic attitude), and Reduced Efficacy (diminishment of one’s accomplishment) (Maslach et al. 1996). It uses a 7-point Likert-type scale (0 = *never* to 6 = *every day*) and has been shown to possess good internal consistency and construct validity (Schaufeli et al. 2002). The alpha coefficients for the three subscales of burnout in the current sample were 0.86, 0.81, and 0.77, respectively.

The UWES is a 17-item self-report scale commonly used to assess work engagement through the three dimensions of Vigor (high levels of mental energy and resilience), Dedication (sense of significance, pride, and enthusiasm), and Absorption (concentration and engrossment in one’s work) (Schaufeli & Bakker 2003). It uses a 7-point Likert-type scale (0 = *never* to 6 = *every day*), and previous research has suggested adequate internal consistency and construct validity (Schaufeli et al. 2002; van Doornen et al. 2009). The alpha coefficients for the three subscales of engagement in the current sample were 0.81, 0.84, and 0.76, respectively.

### Statistical analyses

To analyze the factorial validity of the CPSS-10, we randomly divided the study sample into two subsamples for exploratory (EFA) and confirmatory factor analyses (CFA). We carried out an EFA with the first split sample to explore the scale’s underlying factor structure. We next conducted CFA with the second split sample to test the goodness-of-fit of the revealed factor structures. Table 1 displays the demographic characteristics of the whole sample and the two split samples. Independent t-tests and chi-square tests revealed no significant differences between the two split samples.

We performed EFA on the first split sample using SPSS 19.0, and examined an oblique rotation solution with extraction by principal axis factoring and rotation by oblimin with Kaiser normalization. With a sample size of 491 in the first split sample, the subject-to-item ratio was therefore 49.1:1, well exceeding the recommended ratio of between 5:1 and 10:1 (DeVellis 2003). Sample adequacy was further assessed by the Kaiser-Meyer-Olkin (KMO) measure and Barlett’s test of sphericity. The number of factors was determined with reference to the Kaiser criterion of eigenvalues and the scree test (Costello & Osborne 2005). For the eigenvalue criterion, factors with eigenvalues larger than 1 were kept. For the scree test, we detected the number of factors by locating the last substantial leap in the magnitude of the eigenvalues in the scree plot. We considered factor loadings greater than 0.4 significant. In view of the findings of previous studies (Otto et al. 2004; Ramirez & Hernandez 2007; Reis et al. 2010), we expected either a one- or two-factor solution for the scale.

With the second split sample, we performed CFA using Mplus 5.2 under the maximum likelihood method to examine the goodness-of-fit of a one-factor model and, if applicable, the two-factor model extracted from the EFA with the first split sample. To determine the degree of model fit, we adopted a cluster of criteria on goodness-of-fit statistics: normed chi-square (*χ*^2^/*df*) ≤ 3, a comparative fit index (CFI) ≥ 0.90, a Tucker-Lewis index (TLI) ≥ 0.90, a root mean square error of approximation (RMSEA) ≤ 0.08, and a standardized root mean square residual (SRMR) ≤ 0.06 (Hu & Bentler 1998; Schermelleh-Engel et al. 2003).

We then evaluated the construct validity of the CPSS-10 through examining the bivariate Pearson’s correlations (2-tailed) between its total/factor scores and the validation variables, namely, perceived workload, and the various dimensions of burnout and work engagement. We anticipated the total score of CPSS-10 to be positively associated with perceived workload and burnout, and negatively associated with work engagement. We also examined scale reliability using the Cronbach’s alpha coefficients.

## Results

**Figure 1:**
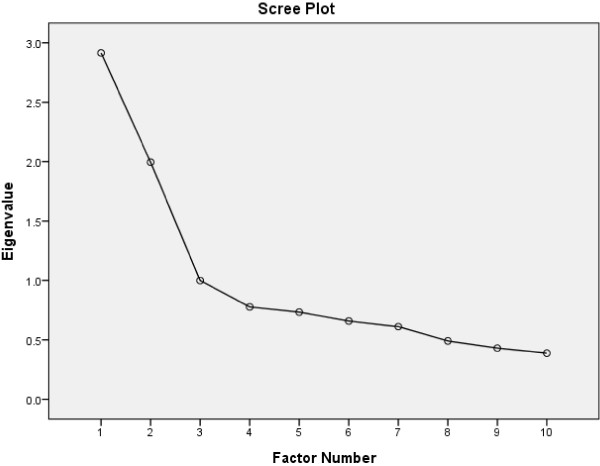
Scree plot of the CPSS-10 in exploratory factor analysis*. * Extraction method: Principal axis factoring; Rotation method: Oblimin with Kaiser normalization.

For the first split sample, the KMO measure was found to be 0.74, while Barlett’s test of sphericity was significant, with *χ*^2^(45) = 1021.61, *p* < 0.01, thus fulfilling the prerequisites for conducting EFA. With regards to the dimensionality of the CPSS-10, the scree plot showed that the curve leveled off after the first two components, with eigenvalues of the two factors greater than 1 (2.92 and 2.00). These findings suggested a two-factor solution for the scale (Figure 1).

Table 2 displays the pattern matrix from EFA with extraction by principal axis factoring and rotation by oblimin with Kaiser normalization. Together the two factors explained 49.10% of the variance. Factor 1 was consisted of six items and accounted for 29.15% of variance, while Factor 2 comprised the remaining four items and accounted for 19.95% of variance. No double loadings occurred in the pattern matrix, with all significant item loadings being greater than 0.5. The two-factor structure revealed was consistent with the factor structure revealed in most previous studies (Orucu & Demir 2009; Otto et al. 2004). Factor 1 was composed of the 6 negatively worded items and Factor 2 was composed of the 4 positively worded items. The Cronbach’s alpha coefficients for Factor 1, Factor 2, and the total score were 0.78, 0.67, and 0.70, respectively. The two factors were weakly correlated with each other (*r* = −0.08, *p* < 0.05).

**Table 2:** Pattern matrix from exploratory factor analysis* of the CPSS-10 in split sample 1 ( ***n*** **= 491)**

		Factor loadings	
PSS items		Factor 1	Factor 2
1	… been upset because of something that happened unexpectedly?	**0.64**	0.07
2	… felt that you were unable to control the important things in your life?	**0.58**	−0.06
3	… felt nervous and ‘stressed’?	**0.61**	−0.05
4	… felt confident about your ability to handle your personal problems?	0.00	**0.50**
5	… felt that things were going your way?	−0.10	**0.57**
6	… found that you could not cope with all the things that you had to do?	**0.52**	−0.07
7	… been able to control irritations in your life?	0.12	**0.64**
8	… felt that you were on top of things?	−0.04	**0.62**
9	… been angered because of things that were outside of your control?	**0.56**	0.07
10	… felt difficulties were piling up so high that you could not overcome them?	**0.73**	0.02
Eigenvalue		2.92	2.00
% of variance explained		29.15	19.95
Total% of variance explained		49.10	
Cronbach’s alpha coefficient of Factor 1 and 2		0.78	0.67
Cronbach’s alpha coefficient of the whole scale		0.70	
Inter-factor Pearson’s correlation (2-tailed)		−0.08	

* Extraction method: Principal axis factoring; Rotation method: Oblimin with Kaiser normalization.

**Figure 2:**
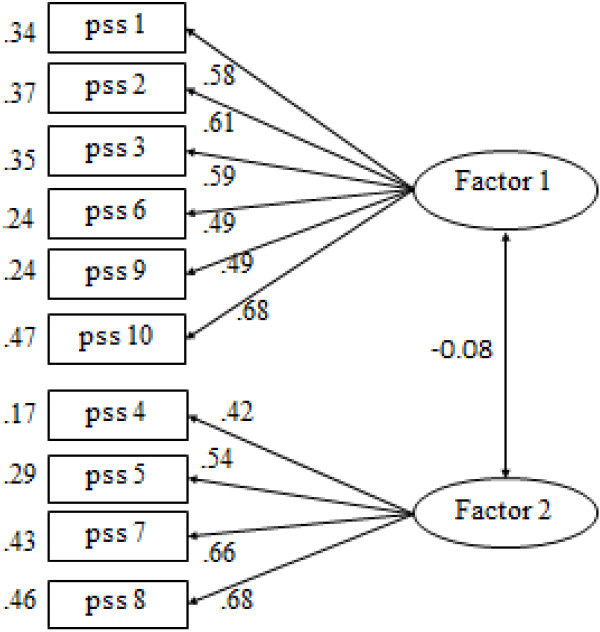
**Two-factor CFA model of the CPSS-10.** All coefficients represent standardized estimates significant at .01 level.

Table 3 displays the goodness-of-fit indices of the CFA models in the second split sample. The one-factor model showed a poor data fit, with *χ*^2^/*df* = 10.09, CFI = 0.62, TLI = 0.52, RMSEA = 0.14, 90% C.I. of RMSEA = (0.12 - 0.15), and SRMR = 0.11. None of the fit indices matched the cutoff criterion. On the other hand, the two-factor model revealed in the EFA showed adequate data fit. The fit indices, given by *χ*^2^/*df* = 2.85, CFI = 0.93, TLI = 0.90, RMSEA = 0.06, 90% C.I. of RMSEA = (0.05 - 0.08), and SRMR = 0.04, were all adequate in accordance with the cutoff criteria. The standardized regression coefficients, as shown in Figure 2, ranged from 0.49 to 0.68 for Factor 1 and from 0.42 to 0.68 for Factor 2.

**Table 3:** **Goodness-of-fit indices of the CFA models of the CPSS-10 in split sample 2 (** ***n*** **= 501)**

Model	χ^2^	df	χ^2^/df	CFI	TLI	RMSEA	CI_90% (RMSEA)_	SRMR
1-factor model	353.07	35	10.09	0.62	0.52	0.14	0.12 - 0.15	0.11
2-factor model	96.86	34	2.85	0.93	0.90	0.06	0.05 - 0.08	0.04

Table 4 presents the Pearson’s correlations (2-tailed) between the CPSS-10 and the validating variables. The total and Factor 1 scores of CPSS-10 showed significant positive correlations with perceived workload (*r* = 0.17 & 0.20), emotional exhaustion (*r* = 0.48 & 0.50), cynicism (*r* = 0.41 & 0.42), and reduced efficacy (*r* = 0.18 & 0.28). Regarding the different dimensions of work engagement, the total and Factor 1 scores of CPSS-10 showed significant negative correlations with vigor (*r* = −0.19 & -0.30), and dedication (*r* = −0.18 & -0.28). For the dimension absorption, only the total score showed significant correlation (*r* = −0.13). The correlation between Factor 1 score and absorption was insignificant.

**Table 4:** **Pearson’s correlations (2-tailed) between CPSS-10 and the validating variables (** ***n*** **= 776)**

Scale		MBI-GS			UWES		
Variable	Perceived Workload	Exhaustion	Cynicism	Reduced Efficacy	Vigor	Dedication	Absorption
Factor 1	0.20*	0.50*	0.41*	0.18*	−0.19*	−0.18*	−0.05
Factor 2	−0.02	−0.16*	−0.18*	−0.25*	0.26*	0.24*	0.17*
Total score**	0.17*	0.48*	0.42*	0.28*	−0.30*	−0.28*	−0.13*

*p < 0.01.

**To compute the total scores, items of Factor 2 were reversely coded and added to items of Factor 1.

In general, Factor 2 score showed correlations with the validation scales in the opposite direction of the total and Factor 1 scores. The Factor 2 score was negatively correlated with exhaustion (*r* = −0.16), cynicism (*r* = −0.18), and reduced efficacy (*r* = −0.25), and positively correlated with vigor (*r* = 0.26), dedication (*r* = 0.24), and absorption (*r* = 0.17). The correlation between Factor 2 score and perceived workload was insignificant.

## Discussion

The PSS-10 intends to be a global and generic scale that measures the degree to which life events are appraised as stressful. Although the Chinese translation of the PSS-10 has been available for over ten years (Lee & Crockett 1994), few studies have closely examined its psychometric properties in samples of Chinese working adults. The existing studies were conducted in a very specific samples, which were cardiac patients who smoked and policewomen (Leung et al. 2010; Wang et al. 2011). The transferability of the findings of these studies is unsure. To our best understanding, the current study is the first study to validate the CPSS-10 in Chinese workers of elderly service.

The EFA with the first split sample revealed an unambiguous two-factor solution with a decent portion of explained variance and a factor loading pattern comparable to results of previous studies (Hewitt et al. 1992; Mimura & Griffiths 2004). Factor 1 (Perceived Helplessness) comprised six negative items, and Factor 2 (Perceived Self-efficacy) comprised four positive items, matching the findings of recent factorial analytic studies on the PSS-10 (Roberti et al. 2006; Reis et al. 2010). The whole scale of the CPSS-10 and Factor 1 Perceived Helplessness showed acceptable levels of internal consistency. With only 4 items, Factor 2 Perceived Self-efficacy showed a relatively lower Cronbach’s alpha coefficient, indicating marginally acceptable consistency.

With the second split sample, CFA revealed a poor fit for the one-factor model suggested by Mitchell, Crane, and Kim (Mitchell et al. 2008). In contrast, the two-factor model revealed in the EFA with the first split sample showed a much better fit, with goodness-of-fit indices meeting all the cutoff criteria.

The current study revealed a weak correlation between Factor 1 and 2 (*r* = −0.08, *p* < 0.05), which is in contrary to previous findings where the two factors were negatively correlated at moderate magnitude. In the study by Leung and her associates (Leung et al. 2010) on 1,860 Chinese cardiac patients who smoked, the inter-factor correlation was revealed to be −0.57. In Wang’s study with 240 Chinese policewomen, the inter-factor correlation was revealed to be −0.47 (Wang et al. 2011). Given the large sample size (992) from multiple sites and high response rate (93%) of the current study, the low inter-factor correlation revealed might suggest a relative independence of the two factors among Chinese elderly service workers. A low, insignificant inter-factor correlation poses some important questions. Are the two factors measuring the same construct? Should a composite score be created for the construct using scores of two uncorrelated factors? Since such low correlation has not been reported in previous studies, the finding should be further tested in different samples, especially with care workers in different social service settings.

With respect to construct validity, we found the CPSS-10 to be associated with various dimensions or manifestations of work well-being as signaled by its convergent property with perceived workload, exhaustion, cynicism and reduced efficacy, and its divergent property with vigor and dedication. The direction of correlations of Factor 1 was generally in line with the total score, whereas Factor 2 showed correlation direction opposite to that of the total score. These results, consistent with research findings on job stress (Duran et al. 2006; Prosser et al. 1997), provide empirical support for the scale’s construct validity in the work context.

Some limitations of the current study must be discussed. The current scale validation study solely relied on self-reported measures. Future studies might incorporate other assessment modalities, such as behavioral appraisals and physiological markers of stress, so as to further substantiate the construct validity. Also, we scrutinized the CPSS-10 in a convenience sample of working adults in the elderly service field. Although there was substantial diversity in their age, marital status, and education levels, over 83% of them were women. This imbalanced gender composition might lower the sample representativeness and limit the generalization of the findings to other occupations where the male-to-female ratio differs substantially from the current study sample. We therefore recommend that further studies be undertaken to evaluate the CPSS-10 in samples with different gender composition. Even so, the large sample size (*N* = 992) and high response rate (93%) of the current study did lend support to the accuracy of the findings. Another notable strength of this study is that we performed scale validation through simultaneous use of EFA and CFA on random split samples. Previous studies have rarely adopted such a cross-validation approach, with the exception of one recent report (Reis et al. 2010).

## Conclusions

In summary, the Chinese version of the PSS-10 (CPSS-10) demonstrated a stable two-factor structure consistent with the findings of most previous studies. It further displayed acceptable internal consistency and adequate evidence for construct validity, and we recommend its use in stress-related research in the Chinese population.

## References

[CR1] Chung KF, Tang MK (2006). Subjective sleep disturbance and its correlates in middle-aged Hong Kong Chinese women. Maturitas.

[CR2] Cohen S, Williamson G, Spacepan S, Oskamp S (1988). Perceived stress in a probability sample of the United States. The social psychology of health.

[CR3] Cohen S, Kamarck T, Mermelstein R (1983). A global measure of perceived stress. Journal of Health and Social Behavior.

[CR4] Cohen S, Tyrrell DA, Smith AP (1993). Negative life events, perceived stress, negative affect, and susceptibility to the common cold. Journal of Personality and Social Psychology.

[CR5] Costello AB, Osborne JW (2005). Best practices in exploratory factor analysis: Four recommendations for getting the most from your analysis. Practical Assessment Research and Evaluation.

[CR6] DeVellis RF (2003). Scale development: theory and applications.

[CR7] Duran A, Extremera N, Rey L, Fernandez-Berrocal P, Montalban FM (2006). Predicting academic burnout and engagement in educational settings: assessing the incremental validity of perceived emotional intelligence beyond perceived stress and general self-efficacy. Psicothema.

[CR8] Eskin M, Parr D (1996). Introducing a Swedish version of an instrument measuring mental stress.

[CR9] Gao LL, Chan SWC, Mao Q (2009). Depression, perceived stress, and social support among first-time Chinese mothers and fathers in the postpartum period. Research in Nursing and Health.

[CR10] Hewitt PL, Flett GL, Mosher SW (1992). The perceived stress scale: factor structure and relation to depression symptoms in a psychiatric sample. Journal of Psychopathology and Behavioral Assessment.

[CR11] Ho RTH, Chan CLW, Ho SMY (2004). Emotional control in Chinese female cancer survivors. Psycho-Oncology.

[CR12] Hu L, Bentler PM (1998). Fit indices in covariance structure modelling: sensitivity to underpararmeterized model misspecification. Psychological Methods.

[CR13] Lazarus RS, Folkman S (1984). Stress, appraisal and coping.

[CR14] Lee S, Crockett MS (1994). Effect of assertiveness training on levels of stress and assertiveness experienced by nurses in Taiwan, Republic of China. Issues in Mental Health Nursing.

[CR15] Lessage FX, Berjot S, Deschamps F (2012). Psychometric properties of the French versions of the perceived stress scale. International Journal of Occupational Medicine and Environmental Health.

[CR16] Leung DYP, Lam TH, Chan SSC (2010). Three versions of perceived stress scale: validation in a sample of Chinese cardiac patients who smoke. Public Health.

[CR17] Maslach C, Jackson SE, Leiter MP (1996). Maslach burnout inventory manual.

[CR18] Mimura C, Griffiths P (2004). A Japanese version of the perceived stress scale: translation and preliminary test. International Journal of Nursing Studies.

[CR19] Mimura C, Griffiths P (2008). A Japanese version of the perceived stress scale: cross-cultural translation and equivalence assessment. BMC Psychiatry.

[CR20] Mitchell AM, Crane PA, Kim YK (2008). Perceived stress in survivors of suicide: psychometric properties of the perceived stress scale. Research in Nursing and Health.

[CR21] Ng SM, Fong TCT, Wang XL (2011). The role of holistic care culture in mitigating burnout and enhancing engagement: A study among elderly service workers in Hong Kong. Aging & Mental Health.

[CR22] Orucu MC, Demir A (2009). Psychometric evaluation of perceived stress scale for Turkish university students. Stress and Health.

[CR23] Otto MW, Fava M, Penava SJ, Bless E, Muller RT, Rosenbaum JF (2004). Life event, mood, and cognitive predictors of perceived stress before and after treatment for major depression. Cognitive Therapy and Research.

[CR24] Prosser D, Johnson S, Kuipers E, Szmukler G, Bebbington P, Thornicroft G (1997). Perceived sources of work stress and satisfaction among hospital and community mental health staff, and their relation to mental health, burnout and job satisfaction. Journal of Psychosomatic Research.

[CR25] Ramirez MTG, Hernandez RL (2007). Factor Structure of the Perceived Stress Scale (PSS) in a sample from Mexico. Spanish Journal of Psychology.

[CR26] Reis RS, Hino AAF, Rodriguez-Anez CR (2010). Perceived stress scale reliability and validity study in Brazil. Journal of Health Psychology.

[CR27] Ro KEI, Tyssen R, Hoffart A, Sexton H, Aasland OG, Gude T (2010). A three-year cohort study of the relationships between coping, job stress and burnout after a counselling intervention for help-seeking physicians. BMC Public Health.

[CR28] Roberti JW, Harrington LN, Storch EA (2006). Further psychometric support for the 10-item version of the perceived stress scale. Journal of College Counseling.

[CR29] Schaufeli WB, Bakker AB (2003). Utrecht work engagement scale: preliminary manual.

[CR30] Schaufeli WB, Salanova M, Gonzalez-Roma V, Bakker AB (2002). The measurement of engagement and burnout: a two sample confirmatory factor analytic approach. Journal of Happiness Studies.

[CR31] Schermelleh-Engel K, Moosbrugger H, Muller H (2003). Evaluating the fit of structural equation models: tests of significance and descriptive goodness-of-fit measures. Methods of psychological research online.

[CR32] van Doornen LJP, Houtveen JH, Langelaan S, Bakker AB, van Rhenen W, Schaufeli WB (2009). Burnout versus work engagement in their effects on 24-hour ambulatory monitored cardiac autonomic function. Stress and Health.

[CR33] Wang Z, Chen J, Boyd JE, Zhang H, Jia X, Qiu J, Xiao Z (2011). Psychometric properties of the Chinese version of the perceived stress scale in policewomen. PLoS One.

[CR34] Wongpakaran N, Wongpakaran T (2010). The Thai version of the PSS-10: an investigation of its psychometric properties. BioPsychoSocial Medicine.

[CR35] The pre-publication history for this paper can be accessed here:http://www.biomedcentral.com/2050-7283/1/9/prepub

